# Dissolved iron elution from mangrove ecosystem associated with polyphenols and a herbivorous snail

**DOI:** 10.1002/ece3.5199

**Published:** 2019-05-29

**Authors:** Ko Hinokidani, Yasuhiro Nakanishi

**Affiliations:** ^1^ Graduate School of Agriculture Tokyo University of Agriculture Tokyo Japan

**Keywords:** detritivorous snail, dissolved iron, mangrove, plant–animal interaction, polyphenols, *Terebralia palustris*

## Abstract

Interest in the systems supplying dissolved forms of iron to the sea from upland forests and wetlands has increased because iron is abundant on land but has low bioavailability in seawater. This can be a limiting factor for the growth of marine phytoplankton. Organic complex iron, a typical form of iron dissolved in seawater, is supplied to the ocean through rivers from forest and wetland soils. As a related study, we focus on mangrove ecosystems located at the boundary between the land and sea and on polyphenols present in leaves as ligands for the formation of iron complexes. When mangrove leaf litterfalls on the wet forest floor, phenolic compounds leach out from the leaves and might solubilize insoluble iron in the sediments (i.e., iron complexation). However, the reaction mechanism is not simple in the field, and it might be made more complex by tidal currents and intervention by crabs and snails, which consume mangrove leaf litter. In the present study, we focused on a detritivorous snail, *Terebralia palustris*, as a facilitator of iron solubilization associated with phenolic compounds, and examined how the snail contribute to iron solubilization processes. Our results indicated that the amounts of phenolic compounds in mangrove sediments are strongly related to iron solubilization. Furthermore, the average dissolved iron and phenolic contents in sediments from areas inhabited by the snail were significantly higher than those of sediments where the snail was not present. We additionally report that the solubilization of iron was promoted when snail feces were added to mangrove sediments. In conclusion, we propose that iron solubilization in mangrove sediments is promoted by the interaction between i) iron in the sediment, ii) phenolic compounds derived from mangroves, and iii) the consumption of leaves and the deposition of feces by the snail.

## 1 INTRODUCTION

Although iron is an abundant metal element in the earth's crust (Clarke & Washington, 1924), iron solubility in seawater is extremely low. This is because inorganic iron speciation in oxic seawater at around pH 8 (Chester, 1990) is dominated by iron hydrolysis behavior, with inorganic iron immediately precipitating as particulate Fe(III) hydroxide (Byrne & Kester, 1976; Millero et al., 1995; Waite, 2001). For this reason, the amount of dissolved iron and its bioavailability in seawater is extremely low. This can be a limiting factor for the growth of phytoplankton, which are the primary producers in marine ecosystems (Martin & Fitzwater, 1988; Martin, 1992; Takeda, 1998). On the other hand, organically bound iron (i.e., organic complexed Fe) is relatively stable in seawater. Some studies have reported the existence of an organic ligand which forms complexes with Fe(III). The majority of iron in seawater exists in the particulate phase, whereas more than 99% of dissolved Fe(III) is bound by organic ligands (Hutchins, Witter, Butler, & Luther, 1999; Rue & Bruland, 1995; Wu & Luther, 1995). In addition, natural organic Fe(III) chelators exist at significant concentrations and control the concentration of dissolved iron in seawater (Kuma, Nishioka, & Matsunaga, 1996). Recently, it has become clear that organically bound iron is supplied to the sea through rivers from forests and wetlands and may contribute to maintaining the growth of phytoplankton in coastal regions (Krachler, Jirsa, & Ayromlou, 2005; Krachler et al., 2010; Matsunaga, Nishioka, Kuma, Toya, & Suzuki, 1998; Nagao et al., 2010). In this context, it has been suggested that humic substances play a key part as organic ligands in iron complexation and iron transportation. In addition, most recent evidence has indicated that several phenolic compounds originating from peatland are also effective transporters of iron from terrestrial systems to the sea (Wu et al., 2016; Wan et al., 2018). However, the mechanism of dissolved iron generation in forests and wetlands is not fully clear.

In tropical and subtropical regions in the world, mangrove forests develop at the boundary between land and sea. In the intertidal zone, organic matter and nutrients produced in mangrove forests are exported to the ocean by tidal activity (Lee 1995). In this context, some studies have reported that tidal transport (outwelling) from mangrove ecosystems contributes to marine productivity (Ayukai, Miller, Wolanski, & Spagnol, 1998; Alongi, Ayukai, Brunskill, Clough, & Wolanski, 1998; Boto & Wellington, 1988; Dittmar et al., 2006). These previous studies have mainly focused on carbon, nitrogen, and phosphorous. In total evaluations of the contribution of mangroves to marine productivity, dissolved iron, one of the major limiting factors of primary oceanic production, is an important element, in addition to the abovementioned nutrients. However, studies of dissolved iron in tropical and subtropical coastal regions are few. Sanders et al. (2015) reported that dissolved iron concentrations in creek waters collected from a creek surrounded by subtropical wetland (mangroves and salt marsh) in Australia were higher than the world average, which suggested that creeks can be an important iron source in the region. However, the elution mechanism of dissolved iron has not yet been clarified.

Within this context, in our previous studies, we found that dissolved iron was eluted from mangrove sediments by mangrove leaf powder, and the eluted amount was significantly positively correlated with the phenolic content in the leaves (Matsutani, Nagai, Kinjyo, & Nakanishi, 2013). This indicated that phenolic compounds can be related to the elution process. Some studies have reported that phenolic compounds, which are secondary metabolites in plant tissues, form organic complexes with metal ions. For example, previous studies (Kennedy & Powell, 1985; Slabbert, 1992) have reported that condensed tannins can form organic metal complexes with iron (i.e., solubilize insoluble iron), and the dihydroxyl group of the flavonoid B‐ring (catecholate) of the condensed tannins is the main group involved in metal complexation (Kennedy & Powell, 1985; Slabbert, 1992). Other studies showed that chlorogenic acid and flavonoids bearing the catechol group also possess iron chelation properties (Andjelkovic et al., 2006; Cesco et al., 2012; Morel et al., 1993). In the ecological context, it is known that iron solubilization by complexation improves iron bioavailability in general soils (Cesco et al., 2012; Colombo, Palumbo, He, Pinton, & Cesco, 2014). For this reason, iron solubilization by phenolic compounds will improve iron bioavailability in sediments, and the solubilized iron may be easily exported to coastal seas by tides. Thus, iron solubilization in mangrove sediments and iron export to coastal seas can contribute to marine primary production in tropical and subtropical regions.

When mangrove leaf litterfalls on wet forest floors, some phenolic compounds leach out from the leaves and might react with iron in the sediment. However, in intertidal zones such as mangrove forests, the reaction mechanism might be more complex. During high tides, the leaf litter can be carried away by tidal currents. Thus, even if phenolic compounds originating from mangrove litter are supplied to the forest floor, tidal activity can prevent the compounds from reacting with iron in the sediments. However, the aerial mangrove root structure can also trap some leaf litter. Leaf‐removing crabs can play a significant role in counteracting the washing out of leaf litter. Regarding to these crabs, many reports have shown that the removal and consumption of leaf litter by detritivorous crabs is an important trophic pathway in mangrove forests, preventing the tidal export of valuable detritus from the ecosystem (Ashton, 2002; Kristensen, 2008; Chen & Ye, 2008; Micheli, 1993; Robertson & Daniel, 1989). In our previous study, the amount of litterfall and its removal by a detritivorous crab, *Neosarmatium smithi*, were investigated in a subtropical mangrove forest in Okinawa, Japan, for 1 year. The crab was found to remove 71, 70, and 37% of the total annual litterfall from *Bruguiera gymnorrhiza*, *Rhizophora stylosa* (mangrove species), and *Derris trifoliata* (nonmangrove species; mangrove associate), respectively (Matsutani, Nagai, & Nakanishi, 2013). Thus, supposing that detritivorous crabs move an amount of mangrove leaf litter to burrows, opportunities for polyphenols in the leaves to react with iron in the mangrove sediment will greatly increase. In this way, by removing mangrove leaves that are rich in polyphenols, detritivorous crabs must play an important role in the polyphenol dynamics in mangrove ecosystems.

**Figure 1:**
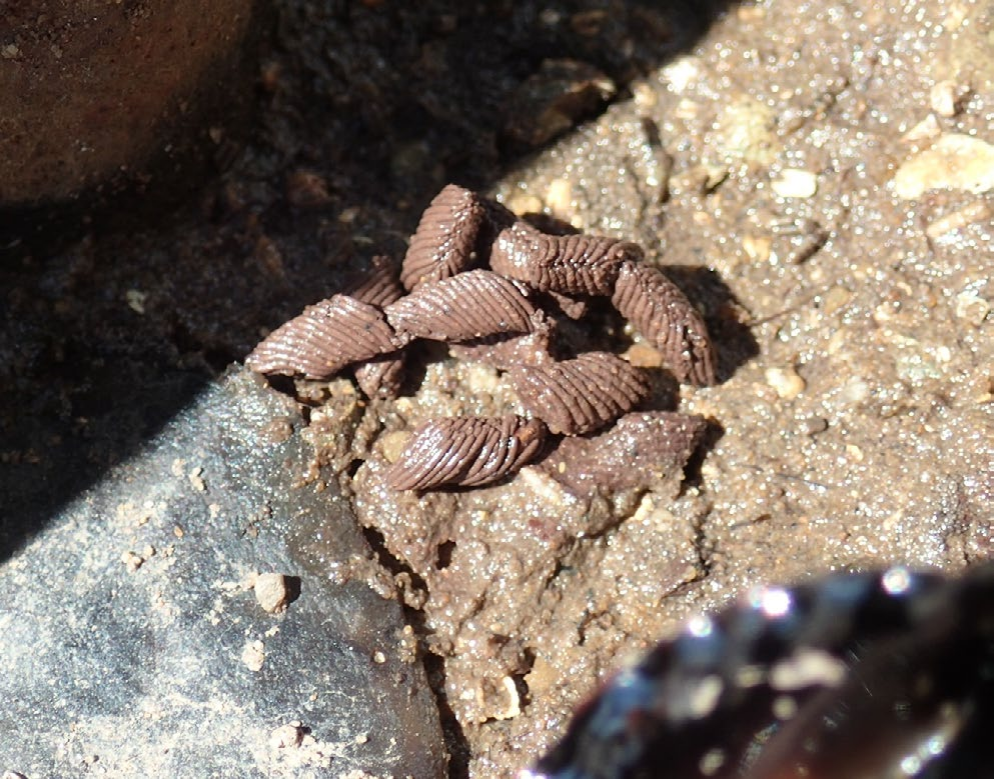
Feces of *Terebralia palustris*

In addition to detritivorous crabs, it is known that *Terebralia palustris* (Gastropoda; Potamididae) inhabits the mangrove forest floor, feeding on mangrove leaves (Fratini, Cannicci, & Vannini, 2000; Fratini, Vigiani, Vannini, & Cannicci, 2004; Slim et al., 1997) and depositing feces on the forest floor (Figure 1). This snail is the only gastropod species that consumes large amounts of mangrove leaf litter in the Indo‐Pacific region (Cannicci et al., 2008). Most of the leaf consumption has been attributed to adult snails (Slim et al., 1997). The highest values of density of the snails have been reported as 150 adults/m^2^ in New Caledonia (Plaziat, 1984). They are thus known to be significant consumers of leaf litter (Fratini et al., 2004; Slim et al., 1997).

Fratini et al. (2004) reported that a total of 10.5 leaves/m^2^ were consumed by adult snails during low tide (6 hr) in a *Rhizophora mucronata* forest in Kenya. This value is equivalent to five times the daily leaf production of *R. mucronata* recorded in another Kenyan mangrove (Slim, Gwada, Kodjio, & Hemminga, 1996).

When consuming mangrove leaves, the snails swarm on the leaves; therefore, this behavior might physically prevent the discharge of leaf litter by tidal currents. Moreover, the easy leaching of phenolic compounds from the leaves caused by damage from eating might promote the solubilization of insoluble iron in the sediment by complexation with phenolic compounds. Furthermore, on forest floors inhabited by *T. palustris*, feces of the snail are deposited on top of the sediment, and surface sediments are often observed to be mixed with the feces. Concerning this point, Carlén and Ólafsson (2002) experimentally demonstrated that the presence of adult snails induces mud surface rearrangement, causing destabilization of the sediments due to the dragging of heavy shells. Thus, if some of phenolic compounds stemming from mangrove leaves remain in the feces, iron solubilization may be promoted by mixing between the mangrove sediments and snail feces. Based on this reasoning, the present study was conducted to determine whether *T. palustis* plays a significant role in dissolved iron elution from mangrove sediment. A further goal was to obtain information on the association of the snails with polyphenols in mangrove leaves consumed as food sources.

First, mangrove sediments where *T. palustris* was present/or not on the forest floor were collected from mangrove forests of three islands in Okinawa, Japan, for measurements of phenolic compound, dissolved iron, and hydrogen ion, and we examined their interrelationships. Second, in order to determine the presence of iron chelators in mangrove leaves and feces of the snail, we identified phenolic compounds in the samples by mass spectrometry.

## 2 MATERIALS AND METHODS

### 2.1 Sample collection

From 2014 to 2015, twenty‐nine mangrove sediments were collected from 13 sites (Funaura bay: *n* = 2; Maira creek: *n* = 2; Shiira creek: *n* = 2; opposite shoreline of Yubu Island: *n* = 2; Kuura creek; *n* = 1; Urauchi river: *n* = 2; Nishida creek: *n* = 1; Hinai creek: *n* = 8; Komi: *n* = 2; Manko: *n* = 1; Kesaji creek: *n* = 2; Nagura creek *n* = 2; Miyara creek: *n* = 2) on three islands: Iriomote Island (Lat. 24°17′N, Long. 123°51′E), Ishigaki Island (Lat. 24°20′N, Long. 124°09′E), and Okinawa Island (Lat. 26°28′N, long. 127°55′E). All sediment samples were collected from the forest floor (0–15 cm depth) of a mangrove forest at each site. The samples of 0–15 cm cover a huge range of environmental conditions (e.g., oxygen, sulfate, redox, and pH ranges). Detailed information on the sediment samples is shown in Appendix S1.

As reference, Kunigami Mahji soil (a red‐yellow soil), which is distributed widely on Iriomote Island, was collected from a pineapple field on the island in 2015, and an andosol (commercially sold as a horticultural soil) was also provided. The samples were air‐dried at 25 ± 5°C, ground with a mortar, and sieved through a 2 mm stainless steel mesh to remove pebbles and organic remains.

When collecting the sediments, we also observed the presence of the detritivorous snail, *T. palustris*, at each sampling site.

Live samples of *T. palustris* (adult snail: shell height, 8–10 cm, *n* = 80) and fresh mangrove leaves were collected from a mangrove forest of *Rhizophora stylosa* and *Bruguiera gymnorrhiza* on Iriomote Island in 2015–2016. The mangrove leaves used for feeding were fresh green leaves. *Terebralia palustris* is known to be one of the major consumers of mangrove leaf litter in the Indo‐Pacific region (Cannicci et al., 2008; Slim et al., 1997). The contribution of other food sources (e.g., microalgae) to snail diets has also become clear through stable isotope analysis (Raw, Perissinotto, Bird, Miranda, & Peer, 2017). Since this study focused on phenolic compounds in mangrove leaves, it was necessary to exclude influences from other food sources. In the collection of feces, first, the snails were placed in a plastic box (containing about a 5 mm layer of seawater to sustain humidity), starved for 48 hr, and allowed to clear their guts before the start of feces collection in the laboratory. After starvation, fresh mangrove leaves (both of *R. stylosa* and *B. gymnorrhiza*) were fed to the snails to collect the feces. To obtain fresh feces, collection was performed within 12 hr after feeding. Obtaining the feces through the above protocol allowed assurance that the feces stemmed from a given mangrove leaf. This starvation protocol was based on a previous study of crabs (Chen & Ye, 2008; Lee, 1997).

Snail feces was air‐dried at 25 ± 5°C, ground into powder with a mortar, and sieved through a 600 μm stainless steel mesh to remove fine impurities. Then, the sample was placed in a vial, capped tightly, and preserved in a cold (at 4°C) and dark place.

The leaf samples used to measure phenolic contents were dried at 50°C for 48 hr with a forced air‐drier and ground into powder with a grinder.

### 2.2 Dissolved iron, phenolic compound, and hydrogen ion contents in sediment and their interrelationships

#### 2.2.1 Hydrogen ion content

The sediment pH was measured using a pH meter, with a sediment‐to‐solution (H_2_O) ratio of 1:2.5 (w/v), and the hydrogen ion content in the sediment was calculated from the pH value. All measurements were performed in triplicate.

#### 2.2.2 Dissolved iron content in the sediment

The dissolved iron content in the sediment samples was measured as described by Matsutani et al. (2013). Each sediment sample was mixed with Milli‐Q water at a ratio of 1:10 (w/v) in a test tube. After shaking the mixture for 1 hr, it was filtered through a 0.22‐μm membrane filter. The filtrate was collected in a 50‐ml Erlenmeyer flask and buffered to pH 3.2 with a 10 M formic acid, 2.4 M ammonium formate buffer solution (0.25 ml/50 ml of sample solution; Nishioka & Takeda, 2000). The amount of dissolved iron in the filtrate was determined using an inductively coupled plasma optical emission spectrometer (ICP‐AES). Reference solutions for ICP‐AES included 100 mg/L of a multielement stock solution (Custom Assurance Standard for ICP). All labware was soaked in a 6 N hydrochloric acid solution overnight to remove trace metals. All measurements were repeated three times. According to Nishioka and Takeda (2000), the iron filtered through the 0.22 μm mesh included the form of iron available to phytoplankton, and the measured forms of iron should include inorganic ions, inorganic complexes, organic complexes, inorganic colloids, organic colloidal particles, biological particles, and nonbiological particles.

#### 2.2.3 Total phenolic content in sediment, leaves, and feces

Sediment samples: To measure the total phenolic content in the mangrove sediment, we first conducted a preliminary experiment to determine the most efficient solvent (70% v/v aqueous acetone, 80% v/v ethanol, or 80% v/v methanol) and extraction method (shaking for 1 hr, ultrasonic treatment for 20 min, or soaking in solvent for 24 hr). Based on this investigation, we found that shaking for 1 hr with 70% acetone was the most efficient method. For extraction, each sediment was mixed with aqueous acetone at a ratio of 1:10 (w/v) in a threaded glass flask. After shaking the mixture for 1 hr, the mixed liquid was centrifuged for 10 min at 2,330 *g* to separate the sediment particles. The supernatant was collected in a glass test tube and kept on ice. For the assay, the supernatant was used to measure the total phenolic content using the Folin–Ciocalteu method. Samples of 1 ml of appropriately diluted extract, 1 ml of 0.2 N Folin–Ciocalteu reagent, and 0.8 ml of sodium carbonate (7.5%, w/v) were introduced into a test tube. This solution was agitated and left for 45 min to allow the reaction to take place and stabilize. A standard solution was prepared in the same way using tannic acid. The absorbance at 725 nm was determined using a spectrophotometer. A calibration curve was generated with the tannic acid solution (range: 1–40 mg/L), and the result was expressed as mg of tannic acid equivalent (TAE) per gram of dry weight. All analyses were performed in triplicate, and the data were used to calculate the mean and for statistical analyses.

Feces and leaves: To measure the total phenolic content in the feces and mangrove leaf samples, we also first conducted a preliminary experiment to determine the most efficient extraction method, as with the sediment samples. Based on this investigation, we judged that ultrasonic treatment with 70% acetone was the most efficient method. The total phenolic contents in the mangrove leaf extract and feces were determined by the Folin–Ciocalteu method (Makker, 2003).

### 2.3 Dissolved iron elution from sediment mixed with feces from *Terebralia palustris*

#### 2.3.1 Amount of dissolved iron eluted from the sediment mixed with feces

To verify the effect on the solubilization of iron in mangrove sediment from the feces of *Terebralia palustris*, we used five mangrove sediments (A‐1, F‐1, K‐2, and M‐1) collected from sites where *T. palustris* was not present (see Appendix S1). As references, two upland‐soil samples were used. The details are as follow. Sample A‐1 was collected from a seaward edge spot of a *Rhizophora stylosa* forest in Funaura Bay, and the pH value was 8.14 ± 0.02. Sample F‐1 was from a riverside edge spot in a *Kandelia obovate* forest in Urauchi river, and the pH was 7.58 ± 0.01. Sample H‐1 was from a forest floor soil, on which the back mangrove, *Heritiera littoralis* (mangrove associate) was the dominant mangrove species, and the pH was 5.23 ± 0.03. Sample K‐2 was from the seaward edge of a *Rhizophora stylosa* forest in Kesaji creek, and the pH was 8.24 ± 0.02. Sample M‐1 was from the seaward edge of a *Rhizophora stylosa* forest in Miyara creek, and the pH was 7.88 ± 0.00. Sample A was a commercially available andosol, with a pH of 5.15 ± 0.01 and was used as a control. Sample F, a Kunigami Mahji soil, was used as another control, and the pH was 4.92 ± 0.03.

Feces from *T. palustris* was mixed with each sediment (or reference soil) sample and Milli‐Q at a ratio of 1:1:10 (w/w/v) in a test tube. After shaking for 1 hr, the mixture was filtered through a 0.22‐μm membrane filter (Membrane Solutions LLC). The filtrate was buffered at pH 3.2 with a 10 M formic acid/2.4 M ammonium formate buffer solution (0.25/50 ml). The amount of dissolved iron in the filtrate was measured in the same manner as described above. All labware was soaked overnight in a 6 N hydrochloric acid solution to remove trace metals. All the experiments were repeated three times. Triplicate data were used to calculate the mean and for statistical analyses.

### 2.4 Identification of phenolic compounds in mangrove leaves and feces

Extraction and purification: Phenolic compounds for HPLC‐PDA‐MS analysis were extracted from leaf powder and feces with an aqueous organic solvent as follows. Each 250 mg sample was extracted with 25 ml of aqueous acetone (70%, v/v), subjected to ultrasonic treatment for 20 min, and centrifuged at 4°C for 10 min. These procedures were repeated twice. The supernatants were filtered through glass microfiber filters (Grade GF/F; Whatman), concentrated using a rotary evaporator under reduced pressure at 50°C, and rinsed with distilled water to give a final volume (50 ml). Using the liquid–liquid extraction (LLE) method, the aqueous solution was then transferred to a separating funnel and re‐extracted with 2 × 50 ml n‐hexane to completely remove lipophilic compounds and with 2 × 50 ml AcOEt to separate the phenolic compounds. The aqueous extracts (aqueous fraction) were concentrated under reduced pressure and then dried with a freezing vacuum dryer and stored in a freezer at –20°C until analyses were performed.

In addition to phenolic compounds, the feces was considered to contain organic substances produced through the digestion by *T. palustris*. Therefore, prior to LC‐MS analysis, in order to obtain concentrated extracts that only contained phenolic compounds from the feces, a solid‐phase extraction (SPE) method was performed as follows. Sep‐Pak C_18_ Plus Short Cartridge (360 mg; Waters) was conditioned with 1 ml of methanol and subsequently equilibrated with 1 ml of pure water. Then, an aqueous solution (50 ml) obtained from the feces was loaded onto the SPE cartridge. The cartridge was washed with pure water, and phenolic compounds were subsequently eluted and collected with 2 ml of aqueous methanol at various concentrations (20, 50, and 80%, v/v) and pure methanol. All of the fractionated and purified extracts were filtered through a 0.22‐μm PTFE membrane filter, and phenolic compounds were then identified using HPLC‐PDA‐MS.

Extraction and purification: Detailed description of analytical condition is described in Appendix S1.

## 3 RESULTS

### 3.1 Dissolved iron, phenolic compound, and hydrogen ion content in the sediment and their interrelationships

**Figure 2:**
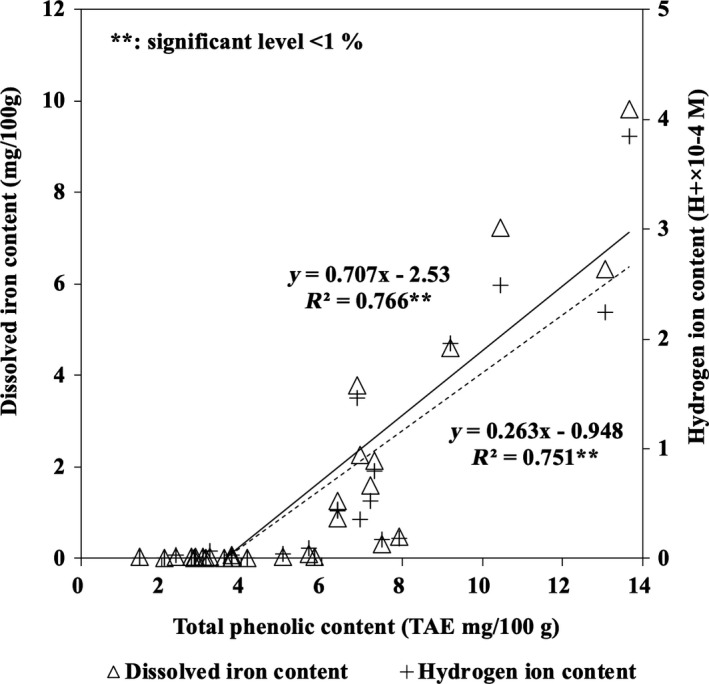
Relationship between total phenolic content and the contents of dissolved iron and hydrogen ions in mangrove sediment samples

As shown in Figure 2, the total phenolic content in the sediment samples expressed in tannic acid equivalents was positively correlated with the contents of dissolved iron and hydrogen ion in the sediment, with correlation coefficients of *R* ^2^ = 0.766 (*p* < 0.001) and *R* ^2^ = 0.751 (*p* < 0.001), respectively. The maximum contents of phenolic compounds and dissolved iron were detected in sample B‐1, at 13.7 ± 0.6 and 9.8 ± 0.4 mg/100 g DW, respectively.

**Figure 3:**
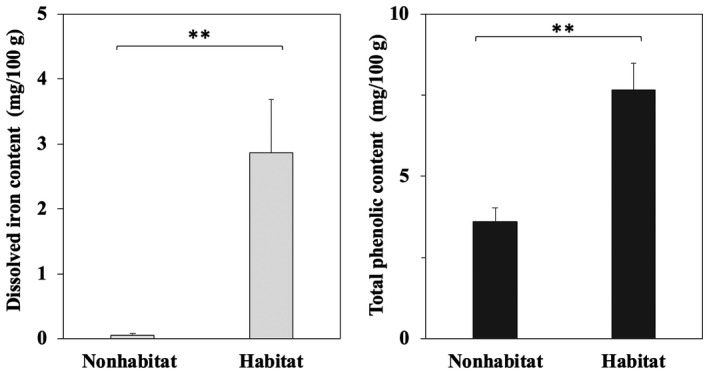
Average levels of dissolved iron and phenolic contents in mangrove sediment samples, arranged based on the existence of *Terebralia palustris*. Habitat (*n* = 14): *T. palustris* inhabited the floor, Nonhabitat (*n* = 15): *Terebralia palustris* was not present. **Significant, *p* < 0.005. Mean ± *SE*

Figure 3 shows the average contents of dissolved iron and phenolic compounds in the mangrove sediment from areas where *T. palustris* inhabited the floor (the habitat sediments, *n* = 14) and in the mangrove sediment from areas where *T. palustris* did not inhabit the floor (the nonhabitat sediments, *n* = 15). The average dissolved iron contents in the habitat sediments and nonhabitat sediments were 0.1 ± 0.1 and 3 ± 3 mg/100 g, respectively. The average phenolic contents in the habitat sediments and nonhabitat sediments were 4 ± 2 and 8 ± 3 mg/100 g, respectively. In both cases, the average values of the habitat sediments were significantly higher than those of the nonhabitat sediments.

### 3.2 Dissolved iron eluted from sediment mixed with *Terebralia palustris feces*

#### 3.2.1 Dissolved iron eluted from sediment mixed with feces

**Figure 4:**
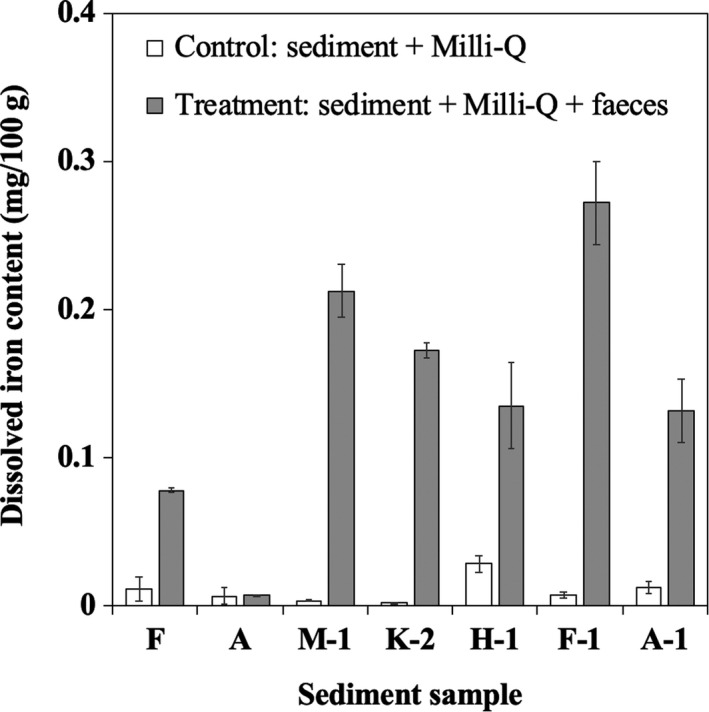
Influence of *Terebralia palustris* feces on dissolved iron elution from sediment and soil samples. F: Kunigami Mahji soil; A: an andosol, A‐1, M‐1, K‐2, F‐1, and H‐1 are mangrove sediments. Mean ± *SD*

As shown in Figure 4, the dissolved iron contents in treated mixtures (sediment or soil + *T. palustris* feces + water) were higher than those in the control mixtures (sediment or soil + water). This result indicated that insoluble iron in mangrove sediments was solubilized by adding snail feces. Particularly with the K‐2 sample, the amount of dissolved iron eluted was 97.5 times greater than that of the control (sediment mixed with water). In addition, although the pH value of *K*‐2 was weakly alkaline, elution was strongly promoted following the addition of feces.

#### 3.2.2 Total phenolic content in feces and mangrove leaves

The total phenolic content in *T. palustris* feces was 6 ± 1 mg/g DW, while that in *R. stylosa* and *B. gymnorrhiza* leaves*,* which were fed to the snail, were 138 ± 14 mg/g and 73 ± 6 mg/g DW, respectively.

### 3.3 Identification of phenolic compounds in mangrove leaves and feces

#### 3.3.1 Mangrove leaves

**Figure 5:**
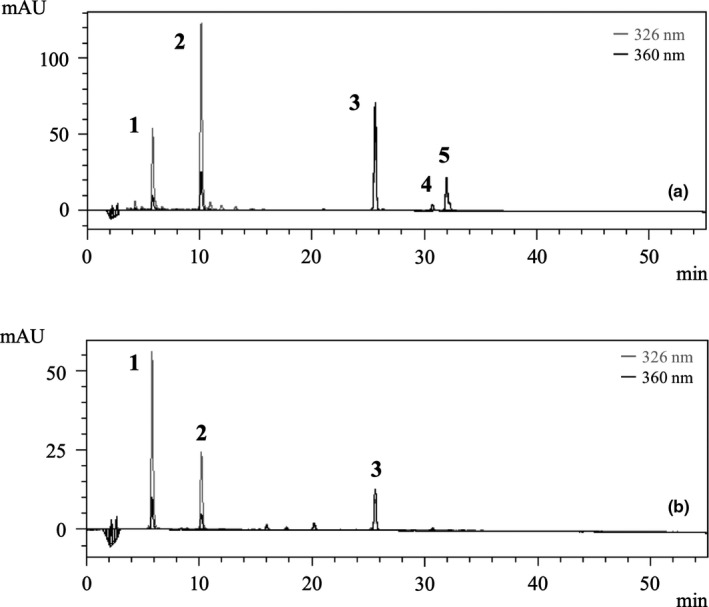
The high‐performance liquid chromatography chromatograms of leaf extracts obtained from (a) *Rhizophora stylosa* and (b) *Bruguiera gymnorrhiza*, using a UV detector at wavelengths of 326 and 360 nm; all peaks were identified using mass spectrometer

Figure 5 shows HPLC‐PDA chromatograms of leaf extracts obtained from (A) *R. stylosa* and (B) *B. gymnorrhiza*, using a UV detector at 326 and 360 nm wavelength. All peaks detected for each extract were identified based on retention time, UV spectra, and mass spectra. The retention time, compound name, UV absorption spectrum, and molecule weight mass are shown in Table 1. The main phenolic compounds in *R. stylosa* leaf extracts were 3‐caffeoylquinic acid (3‐CQA; 5.8 min), 5‐caffeoylquinic acid (5‐CQA; 10.2 min), rutin (25.6 min), kaempferol‐3‐rutinoside (30.7 min), and isorhamnetin‐3‐rutinoside (32.0 min) and from *B. gymnorrhiza* were 3‐CQA (5.8 min), 5‐CQA (10.2 min), and rutin (25.6 min). Peaks representing 3‐CQA, 5‐CQA, and rutin were detected from both extracts of *R. stylosa* and *B. gymnorrhiza*.

**Table 1:** Tentative identification of the main polyphenols observed in leaf extracts obtained from (a) *Rhizophora stylosa* and (b) *Bruguiera gymnorrhiza*, using liquid chromatography coupled with mass spectrometry

Peak	RT min	Compound name	HPLC‐PDA	*m/z*
			λmax	[M‐H]^‐^
(a)				
1	5.8	3‐caffeoylquinic acid	217, 323	353.1
2	10.2	5‐caffeoylquinic acid	217, 326	353.1
3	25.6	Rutin	203, 255, 353	609.1
4	30.7	Kaempferol‐3‐rutinoside	234, 266, 344	593.1
5	32	Isorhamnetin‐3‐rutinoside	203, 253, 353	623.1
(b)				
1	5.8	3‐caffeoylquinic acid	217, 323	353.1
2	10.2	5‐caffeoylquinic acid	218, 329	353.1
3	25.6	Rutin	203, 254, 352	609.1

#### 3.3.2 Feces from *T. palustris*

**Figure 6:**
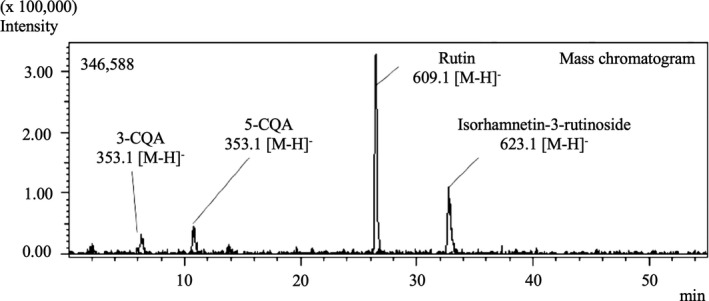
Mass chromatogram of extract obtained from the feces of detritivorous benthos, *Terebralia palustris*, and identification of phenolic compounds in its feces. All peaks were identified using liquid chromatography coupled with mass spectrometry

Figure 6 shows the HPLC‐PDA chromatograms of phenolic compounds contained in feces from *T. palustris* and their identification in 50% MeOH. Regarding the MeOH concentration for SPE extraction, 50% was found to be the most suitable to elute phenolic compounds. All peaks were identified using LC‐MS. Interestingly, five different phenolic compounds were detected in the feces; 3‐caffeoylquinic acid (3‐CQA), 5‐caffeoylquinic acid (5‐CQA), rutin, kaempferol‐3‐rutinoside, and Isorhamnetin‐3‐rutinoside, in addition to the mangrove leaf samples fed to the snail.

## 4 DISCUSSION

### 4.1 Dissolved iron, phenolic compounds, and hydrogen ion content in the sediment and their interrelationships

It is known that Fe(III) oxides (e.g., crystalline Fe and hydroxide) and iron sulfides (FeS and FeS_2_) are the principal forms of iron in mangrove sediments, and these forms are controlled primarily by sulfate reduction and sediment oxidation reactions (Burton, Bush, & Sullivan, 2006; Holmer et al., 1994; Luther, Giblin, Howarth, & Ryans, 1982; Sherman, Fahey, & Howarth, 1998). These forms of iron are insoluble and not bioavailable. In general, iron solubilization is attributed to (a) acidification, (b) reduction, and (c) chelation. (a) Acidification increases the solubility of ferric salts and their bioavailability. Under acidic conditions (~pH 3.5), the concentration of Fe(III) generated from chemical equilibrium is ~10^−9^ M, whereas at higher pH values (~pH 8.5), this concentration decreases to 10^−24^M (Paul, 2015). Thus, even if the Fe(III) in mangrove sediment is solubilized temporarily by acidification, it cannot exist in alkaline solutions such as tidal waters. It will be immediately oxidized and, finally, precipitated as unavailable forms. (b) Oxidation and reduction reactions also play a key part in iron cycling in soils and sediments. Fe(II) is rapidly chemically oxidized to Fe(III) at neutral or alkaline pH values. In this context, it is well known that phenolic compounds have reduction potential toward Fe(III). Upon the binding of a catecholate or gallate ligand to Fe(III), the phenolic compound can reduce Fe(III) to Fe(II) **(**Perron & Brumaghim, 2009 **)**. In (c) chelation, several organic ligands which can bind and solubilize iron have been shown in previous works—microbes release siderophores, and plants exude organic ligands (i.e., phytosiderophores, organic acids, and phenolic compounds). These interactions with plants, microbes, and organic ligands can increase the bioavailability of iron for plant growth (Colombo et al., 2014). Among these interactions, we focused on phenolic compounds in leaf litter as iron chelators. The detailed mechanisms of iron binding with phenolic compounds have been reviewed by Perron and Brumaghim (2009). According to this review, metal ions that prefer octahedral geometry, such as Fe(II) and Fe(III), can be coordinated by up to three catecholate or gallate groups. Moreover, since phenolic compounds are so structurally varied and because the complexes formed are pH‐dependent, the coordination models (the ratio of iron to phenolic compounds) are variable. For example, regarding the catecholate ligand, mono‐complexes—one catecholate ligand per metal ion of iron—predominate under acidic pH (<4) conditions. Under slightly acidic pH (5–6.5) conditions, bis‐complexes—two catecholate ligands per metal ion—predominate, and under alkaline pH conditions, tris‐complexes—three catecholate ligands per metal ion of iron—predominate.

In the present study, to clarify our hypothesis that "polyphenols derived from mangroves can be related with the solubilization process of iron on the forest floor," we examined the relationship between the phenolic content and dissolved iron content in mangrove sediments. The results indicated a significant positive correlation among these contents, and this relationship in mangrove sediments is reported for the first time in this study (Figure 2). This relationship means that more phenolic compounds supplied to the mangrove sediment should promote dissolved iron generation in the sediment. It was also found that dissolved iron could be generated at higher amounts when the level of phenolic compounds exceeded 7 mg per 100 g of sediment. These results strongly indicate that phenolic compounds in mangrove sediment may be related to the solubilization of iron in the sediment.

The relationship between the phenolic content and hydrogen ion content in mangrove sediments (Figure 2) can be explained as follows. Phenolic compounds have higher acidity than alcohol, and their acidic properties stem from their hydroxy groups. When a metal binds with phenolic compounds (i.e., through complexation), the functional group (e.g., a catechol or pyrogallol derivative) is deprotonated, and a hydrogen ion is released at this time. For this reason, it is assumed that phenolic compounds release hydrogen ions when dissolved in water and/or complex with metals. This means that more phenolic compound supplied to mangrove sediment should increase the hydrogen ion content (i.e., decrease the pH value) in the sediment. This pH change in the sediment would change the coordination models of the iron complexed with phenolic compounds.

In general, since mangrove sediment contains more water than does normal upland forest soil, it takes at least one week to air‐dry the sediment samples. Easily decomposable organic substances, such as organic nitrogen compounds, will be decomposed gradually through the air‐drying process. Thus, it is necessary to note that phenolic compounds might also be decomposed or undergo structural changes in the same manner when undertaking a more detailed analysis of phenolic compounds in sediment.

### 4.2 Can mangrove snails promote iron solubilization?

Next, to clarify our hypothesis that “*Terebralia palustris* can promote iron solubilization in mangrove sediment,” we conducted an experiment focused on the existence of the snail and the influence of its feces on iron solubilization in mangrove sediments. As shown in Figure 3, the average phenolic content in snail habitat sediment (*Terebralia palustris* inhabiting the floor, *n* = 14) was significantly higher than that in the nonhabitat sediment (*T. palustris* not present, *n* = 15). This result suggested that the snails have some effect on the accumulation of phenolic compounds in mangrove sediments. This might be through induced generation of dissolved iron by complexation with phenolic compounds in mangrove sediment. In regard to this point, since the average dissolved iron content, as well as phenolic compound content, in the habitat sediments were significantly higher than those in the nonhabitat sediments, this suggested that the snail had some positive effects on dissolved iron generation in mangrove sediment. This result may be explained as follows. When the snails consume mangrove leaves, they swarm on the leaves. This may physically prevent the discharge of leaves by tidal currents, and the easy leaching of phenolic compounds from the leaves caused by "eating" may promote the solubilization of insoluble iron in mangrove sediment by reduction and/or complexation involving the phenolic compounds. Second, if some phenolic compounds stemming from mangrove leaves remain in the feces, they will promote the above chemical reactions (i.e., solubilization) with insoluble iron in the mangrove sediment. Surface sediments mixed with snail feces are often observed in mangrove forests where the snail population is dense. Thus, we considered that if the snail feces contain phenolic compounds, iron solubilization may be promoted by mixing between the mangrove sediments and snail feces.

Regarding the influence of feces on dissolved iron elution, we examined this process using mangrove sediments from areas where *Terebralia palustris* was not present on the forest floor. According to the results, mixing five mangrove sediments with *T. palustris* feces strongly promoted the elution of dissolved iron from the sediments (Figure 4). This result indicated that chemical components in the snail feces had an effect on iron solubilization in the sediment. Based on these three effects on iron solubilization (as mentioned in the above section), the existence of Fe(II) and Fe(III) in the sediment solution could not be assumed. Because the experiment was performed by shaking the solutions to mix the sediment and feces, Fe(II) would be easily oxidized in this process. Since Fe(III) solubility strongly depends on the pH value with the pH range of 7.5–8.5, the solubility reaches a minimum near 10^‐10^ M (Lindsay & Schwab, 1982). Four mangrove sediments had neutral or alkaline pH values (pH 7.6–8.2). Under such pH conditions, the solubility of Fe(III) is extremely low. However, Fe(III) solubility can be improved by organic complexation (Cesco et al., 2012; Colombo et al., 2014). For this reason, we proposed that the forms of dissolved iron generated by adding feces should be organically bound iron. In regard to this point, it is well known that several phenolic compounds can solubilize insoluble Fe(III) by complexation (Cesco et al., 2012). Since phenolic compounds were contained in the feces of the snail, the phenolic compounds in the feces may be related to iron complexation. Thus, in the next experiment, we carried out a detailed analysis of the phenolic compounds in the feces.

### 4.3 Phenolic compounds in mangrove leaves and the feces of mangrove snails

According to the results of LC‐MS analysis, five main phenolic compounds were present in the mangrove leaf samples: P1) 3‐caffeoylquinic acid (neochlorogenic acid), P2) 5‐caffeoylquinic acid (chlorogenic acid), P3) quercetin‐3‐rutinoside (rutin), P4) kaempferol‐3‐rutinoside, and P5) isorhamnetin‐3‐rutinoside. Regarding the *T. palustris* feces, an extract of the snail feces obtained by the LLE method contained high levels of nonphenolic compounds, more than those found in the mangrove leaf samples. Thus, it was necessary to remove the nonphenolic compounds from the extracts. In this case, the use of the SPE method subsequent to the LLE method was found to be suitable for the analysis of phenolic compounds in snail feces. The results showed that four phenolic compounds (except for P4) also contained in mangrove leaf samples were detected in the feces. From these results, we consider that the phenolic compounds in the feces stemmed from the mangrove leaves fed to the snails. The reasoning for this assumption is as follows. Since the *T. palustris* were starved for 48 hr to clear the gut before the collection of feces, the phenolic compounds in the feces should have stemmed from mangrove leaves fed to the snails. Raw et al. (2017) report an exponential decline in the gut pigment content of *T. palustris* over 3 hr. Thus, by starvation for 48 hr, we can exclude the possibility that the phenolic compounds in the feces stemmed from previous food sources.

Two phenolic acids (P1 ~ 2) and three flavonoids (P3 ~ 5) detected in mangrove leaf samples and snail feces have catechol groups in their structures and can form organic complexes with iron (Andjelkovic et al., 2006; Cesco et al., 2012; Morel et al., 1993). Thus, regarding the result shown in Figure 5, we suggest that iron solubilization in the mangrove sediment samples was promoted by these phenolic compounds in the feces, which acted as one of the iron chelators. In addition, our previous study (Matsutani et al., 2013) showed that dissolved iron was eluted from mangrove sediment following the addition of water extracts from *R. stylosa* and *B. gymnorrhiza* leaves. This iron solubilization by the leaf extracts was thought to be because phenolic compounds in the mangrove leaves reacted with the insoluble iron in the sediment and formed chelates (i.e., organic complexes). However, the types of phenolic components in the leaf extracts that reacted with iron were not clarified in the previous study. Based on the results of the present study, we suggest that the chelating agents in the water extracts may have been chlorogenic acids and flavonoids.

Although we only focused on phenolic compounds as iron chelators in the present study, other organic ligands which can bind with iron have been found in various environments (e.g., siderophores, phytosiderophores, organic acids, and humic substances; see Table 2). Among them, Popp (1984) reported that malate and citrate were present in the leaves of several mangrove species. These organic acids have been shown to bind iron, forming mono‐complexes (Jornes, 1998); therefore, we suggest that further studies including these organic acids are needed to clarify the mechanism of dissolved iron elution from mangrove sediments.

**Table 2:** Examples of natural organic chelator on iron solubilization

Ligand type	Example	Origin	Reference
Siderophore	Enterobactin Ferrichrome	Microbe	Saha, Saha, Donofrio, and Bestervelt (2013)
Phytosiderophore	Mugineic acid	Root exudate (Graminaceous)	Sugiura and Tanaka (1981)
Organic acid	Citric acid Malic acid	Root exudate, plant tissue	Jones (1998
Phenolic substance	Phenolic acid Flavonoid	Plant tissue, root exudate	Wu et al. (2016) Cesco et al. (2012)
Humic substance	Humic acid	Abiotic and biotic reactions in soil	Boyd, Sommers, and Nelson (1981)

### 4.4 Ecological context of iron solubilization

The contents of phenolic compounds in the mangrove sediments were particularly higher (>10 mg/100 g) in sample B‐1, sample H‐4, and sample I‐1 than in the other samples. The contents of dissolved iron in the sediments were also particularly higher (>5 mg/100 g) in the above three samples than in the others. The maximum contents were detected in sample B‐1. The details of the sampling sites are below. B‐1 and H‐4 were collected from a spot in *R. stylosa* forests beside creeks, with *T. palustris* densely present at site B (Maira creek) and site H (Hinai creek) on Iriomote Island, respectively. The forest type was riverine mangrove forest. “Riverine” forest types are floodplains flushed by daily tides, which develop along flowing waters such as tidal rivers and creeks that are flooded by high tides and dry during low tides (Woodroffe, 1992). Another twenty sediment samples were collected from this mangrove forest type. I‐1 was collected from a spot in a *R. stylosa* forest without creeks, with *T. palustris* densely populated at site I (Komi) on Iriomote Island. The forest type was "fringe mangrove forest." Fringe mangrove forests have the typical, classic mangrove zonation pattern (Woodroffe, 1992) and are defined along shorelines with elevations higher than the mean high tide. Fringe mangroves grow as relatively thin fringes along shorelines, and they are directly exposed to tides, sea waves, storms, and strong winds with high energy. Another six sediment samples were collected from this mangrove forest type. The common point between the above three sediments with high elution was that the snails inhabited these areas at a relatively high density compared with all other sampling points. In addition to the snails existing, the population density can be closely related to iron solubilization in the sediment. For example, a high density of snails may physically prevent the washout of leaves and promote mixing between surface sediments and snail feces. Furthermore, leaf eating by a larger number of snails may further promote the leaching of phenolic compounds from mangrove leaves. These relationships among the snail density and iron solubilization in this study are not clear at the moment; however, further studies on this relationship are in progress and will be reported elsewhere in the future.

On the basis of our hypothesis, leaf‐removing crabs may also have some effect on the solubilization of iron by phenolic compounds. Typical crabs which consume mangrove litter are Sesarmidae and Gecarcinidae (Micheli, Gherardi, & Vannini, 1991). In regard to this point, burrowing of leaf‐removing crabs did not exist on the forest floor at any of the sampling points except for at point I‐2, although we may not fully exclude the possibility that the crabs have occasionally visited these sites. Regarding sample I‐2, this sediment sample was collected from a spot in a *B. gymnorrhiza* forest without creeks, at a location where *T. palustris* did not inhabit but where crab burrows (diameter: >5 cm) were present. Leaf‐removing crabs, *Neosarmatium smithi*, were observed at point I‐2. Since the phenolic content in sample I‐2 was relatively high (9 mg/100 g), the leaf‐removing crabs may be related to dissolved iron elution in mangrove sediments. At site I (Komi; samples I‐1 and I‐2 were collected from this site) in Okinawa, the habitat of the leaf‐removing crab, *Neosarmatium smithi*, differed from that of *T. palustris*. The former is present from the central area of the *Bruguiera gymnorrhiza* forest to the landward edge, where the flooding level is low, while the latter is present from the seaward edge of *Rhizophora stylosa* to the central area of the *B. gymnorrhiza* forest, where the flooding level is relatively high. Fratini et al. (2000) reported strong competition for mangrove leaves between snails and crabs. Their habitats were close to each other (distance less than 100 m); however, *T. palustris* and *N. smithi* were suggested to have clearly different microhabitats at this site. Point I‐1 (*T. palustris* habitat, *R. stylosa* forest) is an inundating area during all high tides; therefore, litter removal by tidal currents in this area can be higher than that at point I‐2 (*N. smithi* habitat, *B. gymnorrhiza* forest), which is an inundating area during spring tides. In addition to the inundating level, taking into account the interaction of leaf‐removing crabs at point I‐2, it seemed that the opportunity for reaction between phenolic compounds and iron in the sediment is higher at I‐2 than at I‐1. However, the phenolic compound and dissolved iron contents in the sediment were higher at I‐1 than at I‐2. This result may suggest that the snails prevent the loss of the opportunity for iron solubilization by phenolic compounds attributed to leaf removal by tidal currents; however, this effect could not be fully explained by the little evidence obtained herein. In this comparison between snails and crabs, we need to mention two interactions of leaf‐removing crabs. Skov and Hartnoll (2002) reported that the leaf‐removing crab, *Neosarmatium meinerti*, digs burrows 0.8–1.6 m deep. In this study, sediment samples were collected from 0 to 15 cm depth; thus, the samples may not have covered the effect of the crabs in this experiment. Since the crabs remove leaves from the forest floor and consume them in their burrows, the reactions between the leaf and/or feces chemicals and iron may occur in a deeper sediment layer, not in the surface layer.

## 5 CONCLUSION

**Figure 7:**
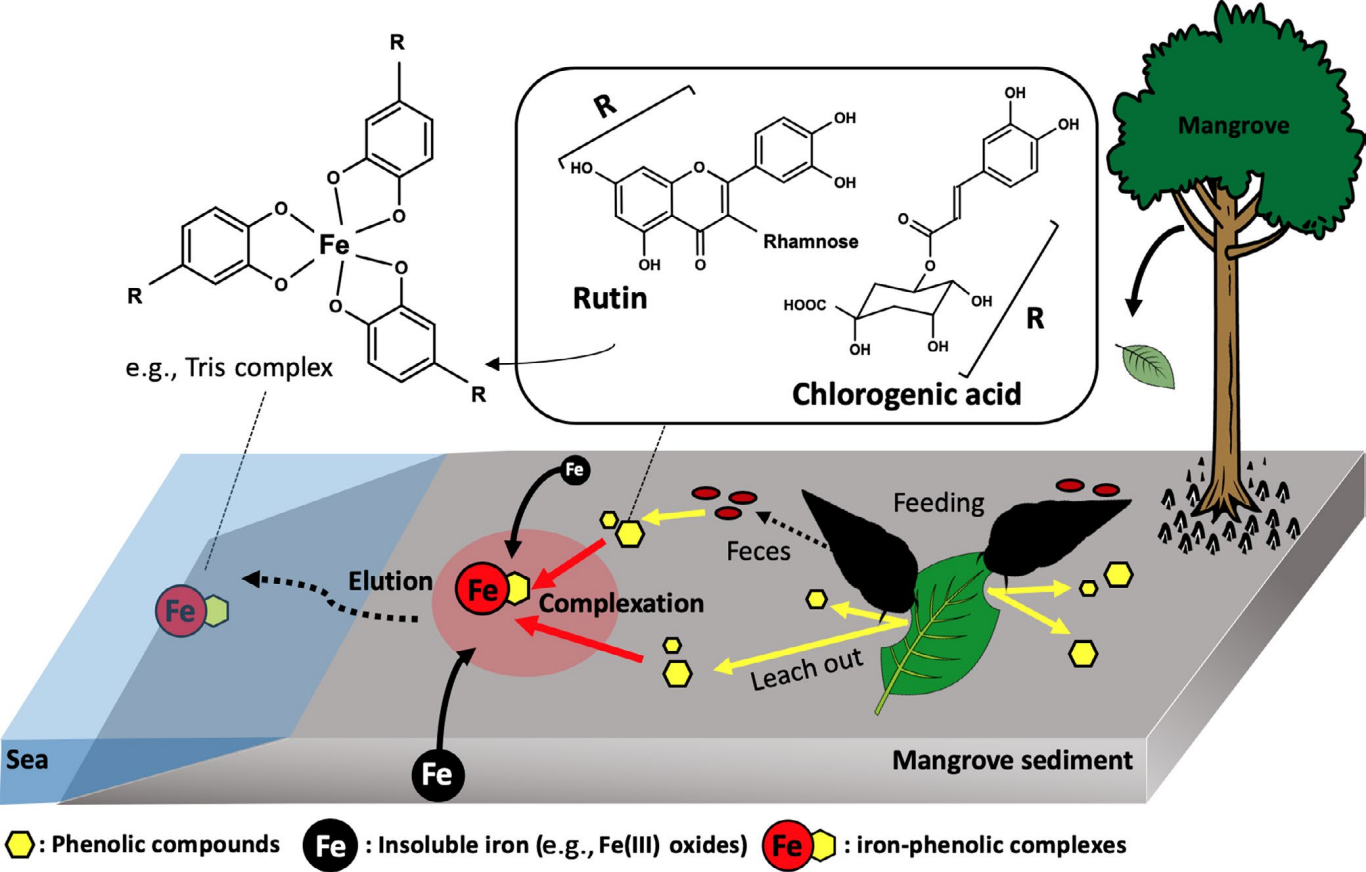
Proposed pathway of dissolved iron elution from mangrove ecosystems to marine ecosystems in a dynamism associated with mangrove‐produced polyphenols, a detritivorous snail, and mangrove sediment

Iron availability is an important factor for photosynthetic organisms and especially for the growth of marine phytoplankton is limited by iron deficiency (Martin & Fitzwater, 1988; Martin, 1992; Takeda, 1998). Recently, it has become clear that coastal wetlands, including mangroves, can play a significant role in the iron supply in subtropical seas (Sanders et al., 2015). However, how the iron is generated in the ecosystem and how the iron is supplied to the sea are not yet clear. In this context, the present study provides the following new findings: (a) the abundance of phenolic compounds in mangrove sediment is strongly related to iron solubilization in the sediments; (b) iron solubilization, which can be attributed to phenolic compounds in mangrove sediments, can be promoted by the intervention of *Terebralia palustris*; and (c) phenolic acids and flavonoids, which can solubilize iron, were detected in mangrove leaves and the feces of *T. palustris*, and these compounds can promote iron solubilization in mangrove sediment by acting as chelators. In conclusion, we propose that iron solubilization in mangrove sediments will be promoted by interaction among (a) iron in the sediment, (b) phenolic compounds derived from mangroves, and (c) the consumption of leaves and deposition of feces by snails (Figure 7). Finally, the population density of snails and the existence of litter‐removing crabs are possibly related to the abundance of phenolic compounds and iron solubilization by phenolic compounds in the sediment. Thus, further studies are needed to fully demonstrate the mechanism of dissolved iron elution from mangrove sediments.

## AUTHORS CONTRIBUTION

YN first designed the study, and KH improved the ideas; KH collected the data; KH analyzed and presented the data; KH led the writing of the manuscript; YN supervised all the progression of the study. All authors contributed critically to the drafts and gave approval for publication.

## Data Availability

All original data used in this study can be found on the Dryad Digital Repository: https://doi.org/10.5061/dryad.md4s537.
