# Supplementary material for: Dissolved iron elution from mangrove ecosystem associated with polyphenols and a herbivorous snail
Source: Ecol Evol. 2019 May 29;9(12):6772–84. doi: 10.1002/ece3.5199 (PMC6662338; doi:10.1002/ece3.5199)
Supplement: Supplementary file 1 [file ECE3-9-6772-s001.docx]

**Appendix 1**

*Detailed description on sediment samples*

**
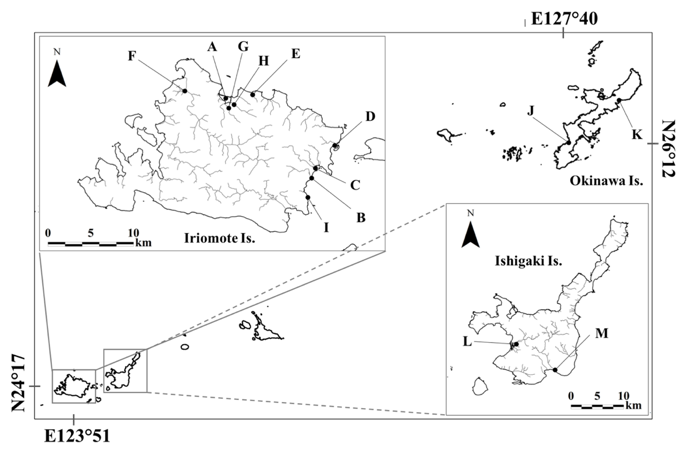
**

**Fig. S1 Sampling sites of this study on Okinawa Is., Ishigaki Is. and Iriomote Is., Japan.**

**Abbreviation.** A: Funaura bay, B: Maira creek, C: Shiira creek, D: Opposite shoreline of Yubu Is., E: Kuura creek, F: Urauchi river, G: Nishida creek, H: Hinai creek, I: Komi; J: Manko, K: Kesaji creek, L: Nagura creek, M: Miyara creek.

**Table S1 Sediment samples and the details.**

**Appendix 2**

*Reagents*

Reagent

The solvents used to extract phenolics from the sediment, leaf, and feces samples were of HPLC grade (Wako Pure Chemical Industries, Osaka, Japan); acetonitrile, methanol, acetone, ethanol, ethyl acetate, and n-hexane. High-purity water refined by a Millipak Express 40 Milli-Q purification system (Merck, Darmstadt, Germany) was used for all analyses and to wash glassware. Folin-Ciocalteu reagent (Sigma-Aldrich Co., St. Louis, MO, USA), sodium carbonate (Wako Pure Chemical Industries), and tannic acid solution (range: 1–40 mgL^-1^, Sigma-Aldrich) were used to measure the total phenolic content in the sediment, leaf, and feces samples. Standard solutions for dissolved iron analysis, were used Custom Assurance Standard for ICP (SPEX CertiPrep, Metuchen, NJ, USA; 5% HNO3). The solvents employed for high-performance liquid chromatography system-coupled electrospray ionization mass spectrometry (HPLC-PDA-MS) analyses were prepared with acetonitrile and formic acid of LC/MS quality (Wako Pure Chemical Industries) and high purity water (Milli-Q).

**Appendix 3**

*Analytical condition on HPLC-PDA-MS system*

Phenolic compounds in the mangrove leaves and snail feces were identified by HPLC-PDA-MS (Shimadzu Co., Kyoto, Japan). The HPLC system was used to separate the compounds and for chromatographic analysis. This system is consisted of a degasser (DGU-20A3), a multi-solvent delivery system (LC-20AB), and auto sampler (SIL-20A), a column holding oven (CTO-20AC), a communicator module (CBM-20A), and photodiode array detectors (SPD-M20A). The mass spectrometry system consisted of a mass spectrometer LCMS-2010EV (Shimadzu Co.) equipped with an ESI source and used to detect molecule mass. Identification analyses were performed in negative ion mode with the following operating MS parameters; interface voltage 4 kV, nebulizing gas flow 1.5 L min^-1^, heating block temperature 200°C, CDL temperature and voltage 250°C and 5.0 V, respectively, Q-Array voltage 0 V DC and 150 V RF, detector voltage 1.5 kV, and scan range m/z 50–2000. Chromatographic separation was achieved on an ODS column: AQ-C18 (3 μm, 150 mm length × 2.1 mm i. d.) reversed-phase column (GL Sciences Co., Tokyo, Japan). Chromatographic conditions were as follows; flow rate 0.2 mL min^-1^, sample injection volume of 5 μL, and mobile phase A: 0.1% (v/v) formic acid in water, and mobile phase B: acetonitrile. The following gradient program was used; 10% B in 0 min, from 10% to 30% B in 50 min, from 30% to 90% B in 55 min, followed by 90% B for 10 min and back to 10% B and 10 min of reconditioning before the next injection. The column oven was set at 40 °C. The detector performed a full spectrum scan between 190 and 800 nm. All data were analysed using LC-MS solution software (Shimadzu Co.). The negative ion mode was used in this study to determine mass due to the better signal-to-noise ratio compared with the positive ion mode.
